# Neurocognitive impairment screening in Romanian HCV infected patients

**DOI:** 10.1186/1471-2334-13-S1-O29

**Published:** 2013-12-16

**Authors:** Ioana-Catrinel Cercel, Șerban Polli, Oana Streinu-Cercel, Anca Streinu-Cercel, Adrian Marinescu, Adrian Streinu-Cercel

**Affiliations:** 1 National Institute for Infectious Diseases “Prof. Dr. Matei Balş”, Bucharest, Romania; 2 Carol Davila University of Medicine and Pharmacy, Bucharest, Romania

## Background

Neurocognitive impairment can occur in HCV infected patients. A series of studies have shown that patients with HCV may experience diminished attention and concentration, or suffer from severe cognitive deficits, such as disorientation and fluctuating consciousness. We performed a study to determine if there are neurocognitive differences or dissimilarities in the quality of life between a control group and a group of HCV infected patients.

## Methods

We assessed patients by applying the EQ-5D-5L questionnaire and the Montreal Cognitive Assessment. EQ-5D-3L comprises the following 5 dimensions: mobility, self-care, usual activities, pain/discomfort and anxiety/depression. Montreal Cognitive Assessment indicates if patients have neurocognitive impairment. All subjects were evaluated with these questionnaires during an appointment with the clinical psychologist.

## Results

We assessed 11 HCV infected patients and 11 controls. The HCV group had ages between 25-58 with a median of 40±12 (2 retired, 1 doctor, 1 economist, 2 engineers, 1 architect, 1 editor, 1 clerk, 1 financial analyst) and the control group had ages between 26-38, median 30±4 (7 doctors, 3 nurses and 1 secretary). EQ-5D-5L scores were 85±10 percent (min: 65, max: 95) in the HCV group and 95±7 percent (min: 85, max: 100) in the control group. The median MOCA scores were 28±4 (min: 19, max: 31) in the HCV group and 30±3 (min: 22, max: 31) in the control group.

## Conclusion

At a first look, we identified apparent neurocognitive differences between the two groups but the number of subjects was too low to offer a statistical significance. We intend to evaluate larger groups of patients, and reapply the tests periodically to determine if there are longitudinal changes in the neurocognitive status.

